# Universal endogenous gene controls for bisulphite conversion in analysis of plant DNA methylation

**DOI:** 10.1186/1746-4811-7-39

**Published:** 2011-12-02

**Authors:** Jing Wang, Chongnan Wang, Yan Long, Clare Hopkins, Smita Kurup, Kede Liu, Graham J King, Jinling Meng

**Affiliations:** 1 National Key Laboratory of Crop Genetic Improvement, Huazhong Agricultural University, Wuhan 430070, P. R. China; 2 Rothamsted Research, Harpenden, Herts., AL5 2JQ, UK; 3 Southern Cross Plant Science, Southern Cross University, Military Road, PO Box 157, Lismore NSW 2480, Australia

**Keywords:** DNA methylation, plants, bisulphite

## Abstract

Accurate analysis of DNA methylation by bisulphite sequencing depends on the complete conversion of all cytosines into uracil. Until now there has been no standard or universal gene identified as an endogenous control to monitor the conversion frequency in plants. Here, we report the development of PCR based assays for one nuclear gene *IND *(*INDEHISCENT*) and two mitochondrial genes, *NAD *(*NICOTINAMIDE ADENINE DINUCLEOTIDE*) and *ATP1 *(*ATPase SUBUNIT 1*). We demonstrated their efficacy as bisulphite conversion controls in *Brassica *and other plant taxa. The target regions amplified by four primer pairs were found to be consistently free from DNA methylation. Primer pairs for *IND.a *and *NAD *were effective within *Brassica *species, whereas two primer pairs for *ATP1 *provided reliable controls across a representative range of dicot and monocot angiosperm species. These primer sets may therefore be adopted as controls in plant methylation analysis for a wide range of studies.

## Background

Methylation of cytosine plays an important role in epigenetic gene regulation in vertebrates and higher plants [1]. In contrast to animals, where methylated cytosine residues are primarily observed within the symmetrical CpG dinucleotide, plants display cytosine methylation in any DNA context, including symmetric CG and CHG (where H = A, T or C) and asymmetric CHH [2]. Over the past few decades, four major approaches have been used for distinguishing the epigenetic mark 5-methylcytosine (5^m^C) from unmethylated cytosine. These include methods based on isochizimer restriction endonucleases, bisulphite conversion of DNA, immunoprecipitation and mass spectrometry [3,4]. Bisulphite conversion of DNA, originally developed by Frommer et al. [5], involves treatment of DNA with sodium bisulphite, where under optimized conditions unmethylated cytosine is converted to uracil, whilst methylated cytosine (both 5^m^C and 5-hydroxymethylcytosine) remains unchanged. DNA sequence changes resulting from bisulphite conversion can then be detected by a variety of methods, including PCR amplification, followed by DNA sequencing where in the original uracil residues are reported as thymine. The primary advantage of this technique is that it provides base-pair resolution of methylation patterns, which is particularly useful in plants for distinguishing between the different cytosine sequence contexts [6]. Following a number of substantial improvements based on the original protocol, bisulphite sequencing is now accepted as the gold standard for detecting changes in DNA methylation [3]. The combination of bisulphite conversion and next-generation high-throughput sequencing has recently provided powerful tools for revealing DNA methylation patterns on a genome-wide scale [7-10].

Although bisulphite-based methods are reasonably accurate and reproducible in comparison with other methods, successful detection is dependent on the complete bisulphite conversion of all unmethylated cytosine into uracil [11]. Incomplete conversion complicates downstream data analysis, especially in plants where larger and more complex genomes are likely to contain a high level of 5^m^C. False-positive 5-methylcytosines (cytosine read as 5-methylcytosine) are common, since it is often difficult to determine whether an unconverted cytosine represents true methylation or incomplete treatment. Both incomplete DNA denaturation prior to bisulphite treatment and reannealing during treatment can lead to incomplete bisulphite conversion, since bisulphite converts single-stranded but not double-stranded DNA [5]. As a result, repeated denaturation cycles during the bisulphite treatment are required to ensure complete conversion [4], which is now a standard feature of protocols recommended for commercial bisulphite conversion kits. However, it is still necessary to include some form of control to monitor bisulphite conversion for each sample assayed. The completion of bisulphite conversion can be tested by monitoring exogenously spiked DNA controls, or retention of endogenous non-target sequence cytosine dinucleotides [4]. Theoretically, any DNA sample with a known consistent methylation pattern could be used as a control. In mammalian genomes, unamplified, nearly methylation-free genomic DNA from specific cell lines has been used as the template to optimize and test conditions for genome-wide bisulphite conversion, PCR amplification and subsequent library construction [8]. In Arabidopsis, specific unmethylated genes and chloroplast DNA have been used for establishing the degree of conversion [9,12,13]. Plant mitochondrial DNA is another potential control for monitoring conversion, since mitochondrial genomes are free of methylated cytosines and can be isolated with nuclear DNA from all organs and tissues [14]. However, to date the full sequence of mitochondrial genomes has only been established for a small number of plant species.

In this study, we first identified a nuclear endogenous gene *IND.a*, present in *Brassica *'A' genomes, which remains unmethylated in different organs and tissues. We then designed primer pairs for two mitochondrial genes, *ATP1 *and *NAD*. Two primer pairs for *ATP1 *were effective across all dicotyledonous and monocotyledonous species tested, and are therefore valuable as universal controls for DNA methylation analysis of target genes or whole genome analysis in plants.

## Results and Discussion

**Figure 1:**
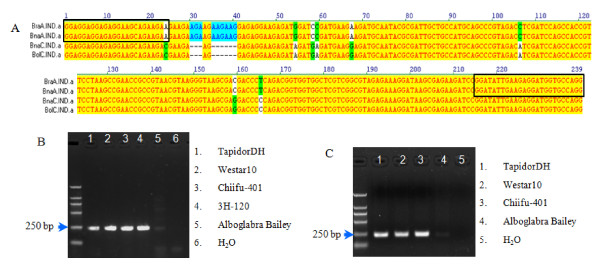
**Diagram of primer design and amplification of IND.a_A3**. (A) The alignment of IND.a_A3 region among *B. rapa*, *B. oleracea *and *B. napus*. The blue open box showed primer region. (B-C) PCR products from IND.a_A3 were abundant in genomic DNA and bisulphite treated DNA from floral bud of *B. napus *and *B. rapa*, however, extremely low in *B. oleracea*. TapidorDH and Westar10 are cultivars of *B. napus*, Chiifu-401 and 3H-120 of *B. rapa*, and Alboglabra Bailey is a *B. oleracea*.

The genus *Brassica *includes a diverse range of important vegetable, oilseed, fodder, and mustard crops grown and consumed throughout the world. It has three widely cultivated diploid species *Brassica rapa *(AA, × = 10), *B. nigra *(BB, × = 8) and *B. oleracea *(CC, × = 9) and three allotetraploid species *B. napus *(AACC, × = 19), *B. juncea *(AABB, × = 18) and *B. carinata *(BBCC, × = 17). *Brassica *genomes are complex, with most genes present as multiple paralogous copies [15-18]. However, only two orthologues of the Arabidopsis *IND *(*INDEHISCENT*) gene were found in the amphidiploid *B. napus*, one within the A genome and one within the C genome [19,20]. We designed a specific primer pair for amplification of *BnaA.IND.a *and *BraA.IND.a*, located on chromosome A3, as a control to monitor bisulphite conversion in *Brassica *species with A genome (Figure 1A). The primer pair IND.a_A3 gave reproducible PCR products when genomic DNA or bisulphite treated DNA was used as a substrate for different *Brassica *species that possess the A genome (Figure 1B and 1C). PCR products from bisulphite treated DNA were cloned and for eight clones selected at random, the sequences demonstrated complete conversion of all cytosine residues to uracil. We therefore conclude that *BraA.IND.a *and *BnaA.IND.a *are consistently free of DNA methylation modification. As such they represent a suitable target for use as a control in DNA methylation analysis for those *Brassica *species possessing the A genome (i.e. *B. rapa *(A), *B. napus *(AC), *B. juncea *(AB)).

In order to develop an assay that would be applicable to all *Brassica *species, we next considered candidate genes within the mitochondrial genome. The orthologues of Arabidopsis *NAD *and *ATP1 *were chosen as suitable targets for developing the control assay due to their potential conservation across plant taxa, and the key role they play in energy production and storage. Two primer pairs for *ATP1 *and one for *NAD *were designed based on a region within the genes conserved amongst different species (Additional file 1). Bisulphite sequencing of eight clones for each treatment indicated complete conversion of cytosine to uracil within the target region of *NAD *for all *Brassica *species. This indicated that, as expected, the gene is free of methylated cytosine. However, it was not possible to amplify this target region of *NAD *from other species. In contrast, although some sequence polymorphism was present in different families of plants (data not shown), the PCR products generated using two primer pairs from the *ATP1 *gene were of identical size (227 bp for ATP1-1 and 252 bp for ATP1-2) in all species tested. Following bisulphite sequencing of eight clones from each treatment, *ATP1 *genes were also found to be universally unmethylated. This result is consistent with the known lack of 5-methylcytosine within mitochondrial genomes [14]. However, the transfer and incorporation of regions of mitochondrial and plastidic genomes within the nuclear chromosomes is relatively common amongst flowering plants [21]. In Arabidopsis, the mitochondrial *ATP1 *gene has been found in the nuclear genome where it is methylated at a low level [13,22]. Detailed analysis of the regions flanked by the two primers ATP1-1 and ATP1-2 indicates that these are free of methylated DNA [13]. Although the methylation status of nuclear *ATP1 *sequences in other plant species is unknown, it appears that the PCR products we generated here are clearly free of 5-methylcytosine, irrespective of their organellar or nuclear origin.

**Figure 2:**
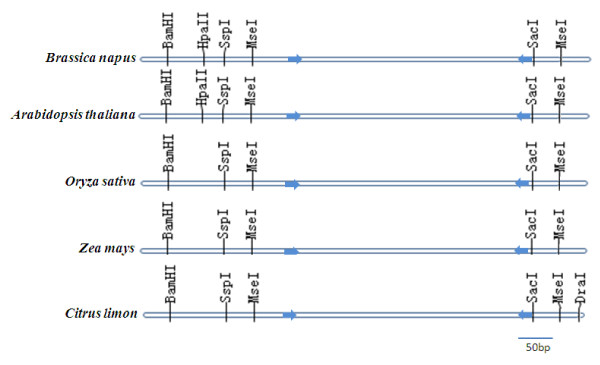
**Schematic representations of restriction endonuclease loci near the target region of ATP1-2 in different species**.

Based on these results, we have identified suitable controls for bisulphite DNA methylation analysis, using PCR amplification of four target regions within three genes. This will facilitate the study of epigenetic variation and regulation of single genes across tissues or environments, as well as genome-wide surveys in different species. In particular, the *ATP1 *primer pairs appear to be valuable as universal controls for the comparison of DNA methylation state across a wide range of plant taxa. Moreover, we identified the presence of specific restriction endonuclease recognition sites within the sequence flanking the ATP1-2 primer pair in different species (Figure 2). This provides an additional application as a useful control for reduced representation bisulphite sequencing (RRBS) in different plant species. RRBS analysis involves fragmentation of genomic DNA by different restriction endonucleases followed by isolation of fragements within a discrete size range, which are then subjected to bisulphite treatment and sequencing [8]. The target region of ATP1-2 will be retained for bisulphite sequencing following digestion with specific endonuclease combination such as *Sac*I and *Mse*I (Figure 2).

**Figure 3:**
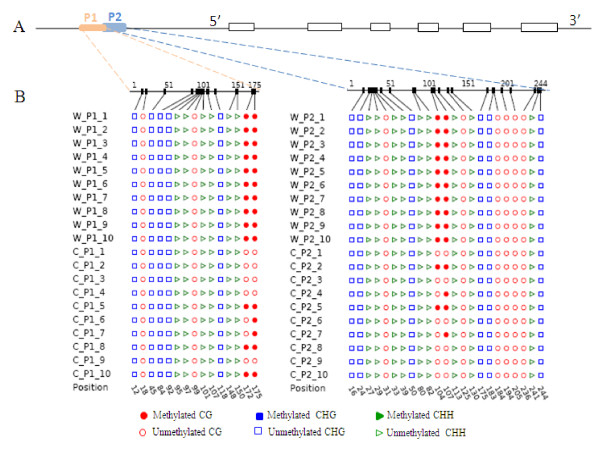
**DNA methylation profiles of *Bra.ATS1 *and *BnaA.ATS1***. (A) Scheme of *Bra.ATS1 *located on chromosome A1 of *B. rapa*. (B) Bisulphite sequencing of two promoter regions, P1 and P2, was performed on DNA collected from floral buds from Westar 10 and Chiifu-401. W represents Westar10 and C represents Chiifu-401. 1-10 designate 10 random clones.

Although our assays appeared not to detect any methylated cytosine for each of the genes we screened, it was important to demonstrate that there had not been excess bisulphite exposure that may have led to conversion of methylated cytosine to uracil. *ATS1 *is a nuclear encoded gene that is specifically transcribed during embryo development in Arabidopsis [23]. The promoter region of *BraA.ATS1 *(which contained a potential cytosine methylation, data not shown) is located on chromosome A1 of *B. rapa*, and was chosen as an additional control to test for the presence or variation of cytosine methylation. Two primer pairs designed from the promoter region were used to generate amplicons from a single source of bisulphite treated DNA isolated from the buds of *B. napus *Westar10 and *B. rapa *Chiifu-401. Although two CG sites were completely methylated in Westar10, the same sites were partially methylated in Chiifu-401 (Figure 3). This suggests that *ATS1 *has a differential methylation pattern at the pre-embryonic stage in the diploid *B. rapa *compared with the amphidiploid *B. napus*. Moreover, the observed variation of cytosine methylation in *B. rapa *may indicate heterogeneity of methylation pattern either in different tissues within the floral bud or reflect different developmental stages. In tomato, the SBP-box gene *LeSPL-CNR *is required for normal ripening, and hyper-methylation within the promoter leads to the "Colorless non-ripening" mutation. Moreover, the methylation pattern varies in different tissues (leaf and fruits), during the stages of fruit development and ripening, and between genotypes [24]. Based on our results, we can be confident that the target regions of *ATP1*, *NAD *and *IND.a *were indeed unmethylated and that the full conversion of cytosine to uracil did not result from excess bisulphite treatment.

We also wished to determine whether two successive bisulphite treatments were required to convert all cytosine to uracil. We therefore repeated the analysis with treated DNA from buds of Westar10 and primer pair ATP1-2, analyzing cloned sequences following each round of bisulphite treatment. We found that there was a significant effect on the conversion frequency following the second round of bisulphite treatment, based on analysis of sequences from 15 clones selected at random from each treatment (Table 1). However, the additional purification following the first round of treatment had no significant effect (Table 1).

**Table 1:** Sodium bisulphite conversion frequencies of four treatments

Treatment	Number of clones	Conversion frequency (%)				
		
		CG	CHG	CHH	Total	t test (P < 0.01)
One round treatment	15	97.57	98.79	98.18	98.18	A
One round treatment + purification	15	96.36	96.97	98.99	98.06	A
Two round treatment	15	99.80	99.39	99.80	99.64	B
Two round treatment + purification	15	99.39	100.00	99.80	99.76	B

We also addressed the potential bias in PCR amplification based on use of the primer sets [25]. Bisulphite treatment converts cytosine to uracil whereas 5-methylcytosine is not converted. Thus the complexity and base composition (Tm) of predominantly unmethylated sequences with unmethylated DNA differ widely before and after treatment. In humans and mice it has been found that most of the primer sets are biased to amplify unmethylated DNA with a high content of thymine following bisulphite conversion [25,26]. Since the target regions of the four primer sets tested all appear to be free of methylation, PCR amplification may have resulted in a bias, leading to an inaccurate estimate of the bisulphite conversion ratio. We therefore designed an experiment to explore the bias of the four primer sets as suggested by Warnecke [25]. The results based on SSCP analysis indicated very different bias values for the different primer sets within the same species, and for the same primer in different species (Table 2). The ATP1-1 primer set in *B. napus *(TapidorDH) had the highest bias value (7.81) whilst the ATP1-2 set in rice (Nipponbare) had the lowest bias value (1.98) (Table 2). All primer sets had an average bias value greater than 1, which indicated that all primers were more likely to amplify un-converted DNA. This gives some reassurance that the bisulphite conversion ratio we estimated here is likely to be a reliable estimate, since the primers were not biased to amplify converted DNA.

**Table 2:** Analysis for PCR bias of different primer sets

Primer set	Percentage (%) of PCR products from genomic DNA in TapidorDH/Value of bias				Average value of bias	Percentage (%) of PCR products from genomic DNA in Nipponbare/Value of bias				Average value of bias
				
	**80**^**a**^	**60**^**a**^	**40**^**a**^	**20**^**a**^		**80**^**a**^	**60**^**a**^	**40**^**a**^	**20**^**a**^	
ATP1-1	95/4.75	90/6.00	85/8.50	75/12.00	7.81	95/4.75	65/1.24	60/2.25	45/3.27	2.88
ATP1-2	90/2.25	80/2.67	65/2.79	30/1.71	2.36	85/1.42	70/1.56	60/2.25	40/2.67	1.98
IND.a_A3	95/4.75	85/3.78	85/8.50	40/2.67	4.93	/	/	/	/	/
NAD	95/4.75	90/6.00	85/8.50	70/9.33	7.15	/	/	/	/	/

^a ^The percentage (%) of non-converted DNA before PCR reaction in mixed template DNA.

## Conclusion

In summary, we have developed a series of control assays that are valuable for ensuring accurate analysis of plant DNA methylation following bisulphite treatment. We have developed one assay that may be applied to *Brassica *species containing the A genome, three that may be applied to all *Brassica *species, and two that appear to be universally applicable to a wide range of monocotyledon and dicotyledon plant taxa. We provide the primer sequences, expected length and number of cytosine residues in the control assays (Table 3).

**Table 3:** Description of six primer sets tested and the resultant PCR products

Primer pair	Forward primer sequence (5'→3')	Reverse primer sequence (5'→3')	Tm (°C)	Expected size (bp)	No. of cytosine in target region
IND.a-A3	GGAGGAGGAGAGGAAGYAGAAGAA	CCTRRCACCATCCTCTTCAATATCC	58, 58	239	43
ATP1-1	TGAAYGAGATTYAAGYTGGGGAAATGGT	CCCTCTTCCATCAATARRTACTCCCA	50, 56	227	64
ATP1-2	TAGTAAAYAGGYGGTGGYATATYGA	CTCTRTTTCCAAACARATTTRTCCATC	50, 56	252	15
NAD	AGTTTYTGYTAGAYGAGAAATAAGGA	CCTACTCACTCRRACAATRCTCT	50, 56	276	24
ATS1-P1	AGGTTYAGGGTTTTGGTAGTGAGAAGGGA	TCCATRACAATCCTAACAACAATTATCA	51, 54	305	54-58
ATS1-P2	TGGAGGAGYAGAGGYGAAGYTTGA	ACCAARACCCRCCACAACACATRCCT	55, 64	227	24-28

## Materials and methods

### Plant materials

Twenty five plant species and synthetic species representing ten genera within six dicot and monocot families were used. DNA was isolated mostly from the leaves, as well as from floral buds and siliques, from plants grown in field or controlled environment conditions (Table 4).

**Table 4:** Plant materials used in this research

Family	Genus &Species	Accession	Tissue	Environment/Location	Development stage	Gene detection
Brassicaceae	*Brassica rapa*	Chiifu-401	Bud	Field (HAU)	Budding	IND.a, ATP1, NAD
			Silique (4 cm)	Field (HAU)	Silique setting	IND.a, ATP1, NAD
		3H-120	Leaf	Field (HAU)	Seedling	ATP1, NAD
	*Brassica oleracea*	Alboglabra Bailey	Bud	Field (HAU)	Budding	ATP1, NAD
			Silique (4 cm)	Field (HAU)	Silique setting	ATP1, NAD
	*Brassica nigra*	Giebra	Leaf	Field (HAU)	Seedling	ATP1, NAD
	*Brassica napus*	TapidorDH	Bud	Field (HAU)	Budding	IND.a, ATP1, NAD
			Silique (4 cm)	Field (HAU)	Silique setting	IND.a, ATP1, NAD
			Leaf _2	CE RRes	Seedling	IND.a, ATP1, NAD
			Leaf _6	CE RRes	Seedling	IND.a, ATP1, NAD
			Leaf_9	CE RRes	Seedling	IND.a, ATP1, NAD
			Leaf_14	CE RRes	Seedling	IND.a, ATP1, NAD
		Westar10	Bud	Field (HAU)	Budding	IND.a, ATP1, NAD
			Silique (4 cm)	Field(HAU)	Silique setting	IND.a, ATP1, NAD
		HC^1^	Leaf	Field (HAU)	Budding	ATP1, NAD
		CH-2^1^	Leaf	Field (HAU)	Budding	ATP1, NAD
	*Brassica juncea*	Hn^2^	Leaf	Field (HAU)	Budding	ATP1, NAD
	*Brassica carinata*	NC^3^	Leaf	Field (HAU)	Seedling	ATP1, NAD
Malvaceae	*Gossypium hirsutum*	Lizhongmian-1	Leaf	Field (HAU)	Seedling	ATP1,
	*Gossypium herbaceum*	Licaomian-1	Leaf	Field (HAU)	Seedling	ATP1,
Fabaceae	*Glycine max*	Zhongdou30	Leaf	Field (OSR)	Flowering	ATP1,
Rutaceae	*Citrus unshiu Marcow*	Guoqing No.1	Leaf	Field (HAU)	Fruit setting	ATP1,
	*Citrus grandis Osbeck*	HB pummel	Leaf	Field (HAU)	Fruit setting	ATP1,
	*Citrus sinensis Osbeck*	Newhall Navel orange	Leaf	Field (HAU)	Fruit setting	ATP1,
	*Citrus limon Burm.f*	Eureka lemon	Leaf	Field (HAU)	Fruit setting	ATP1,
	*Citrus reticulata Blanco*	'Egan No.1'	Leaf	Field (HAU)	Fruit setting	ATP1,
Solanaceae	*Solanum lycopersicum*	Micro Tom	Leaf	Glasshouse (HAU)	Flowering	ATP1,
	*Solanum lycopersicum*	M82	Leaf	Glasshouse (HAU)	Flowering	ATP1,
	*Nicotiana tabacum*	Li yancao-1	Leaf	Field (HAU)	Seedling	ATP1,
Poaceae	*Triticum aestivum*	Huahui8	Leaf	Field (HAU)	Seedling	ATP1,
	*Zea mays*	Mo17	Leaf	Field (HAU)	Booting	ATP1,
	*Oryza sativa ssp. japonica*	Nipponbare	Leaf	Field (HAU)	Seedling	ATP1,
	*Oryza sativa ssp. indica*	Zhenshan97	Leaf	Field (HAU)	Seedling	ATP1,
	*Oryza rufipogon*	Griff.	Leaf	Field (HAU)	Seedling	ATP1,

1, 2, 3 Three *Brassica *synthetic tetraploids. The diploid parents are: *B. rapa *(AA): accession no. 3H-120; *B. oleracea *(CC): *B. alboglabra Bailey*; *B. nigra *(BB): *B. Giebra*.

HAU: Huazhong Agricultural University, Wuhan, China; OSR: Oilseed Crop Research Institute Chinese Academy of Agricultural Science, Wuhan, China; RRes: Rothamsted Research, Harpenden, Hertfordshire, UK

### Bisulphite sequencing

Collection of plant material, storage and DNA extraction followed the DNeasy Plant Mini Kit (Qiagen) handbook. 450-750 ng genomic DNA was subjected to two successive treatments of sodium bisulphite conversion using the EpiTect Bisulphite kit (Qiagen) according to the manufacturer's instructions. The reaction was then purified once more using the PCR purification kit (Qiagen). Forward (F) and reverse (R) primers for bisulphite sequencing PCR were designed using Kismeth http://katahdin.mssm.edu/kismeth based on the reference sequences in GenBank (Additional file 1).The bisulphite-treated DNA was amplified using Maxima™ Hot start Taq DNA polymerase (Fermentas). The thermal cycling program was 95°C for 4 min followed by 35 cycles of 95°C for 30 s, annealing for 30 s, and extension at 72°C for 45 s, ending with a 10 min extension at 72°C. PCR products were cloned into the pMD18-T vector (TaKaRa), and 8-15 individual clones were sequenced. Percentage methylation (% C) was calculated as 100 ×C/(C + T). DNA cytosine methylation in the CG, CHG, and CHH contexts was analyzed and displayed using CyMATE [27].

### PCR bias analysis

Un-converted genomic DNA, either from TapidorDH leaf 9 (dicotyledon) or Nipponbare seedling leaf (monocotyledon), was diluted to the same concentration as bisulphite treated DNA. Four sets of samples were prepared with 80:20, 60:40, 40:60, 20:80 ratios of genomic to bisulphite treated DNA. PCR products generated from these templates with different primer combinations were cloned into the pMD18-T vector (TaKaRa). Twenty individual clones from each primer set were selected randomly for SSCP (single strand conformation polymorphism) analysis to discriminate different products from genomic DNA and bisulphate treated DNA. A value for the bias (b) was calculated as b = [y(100 - x)]/[x(100 - y)]; y is the percent of PCR products from genomic DNA and x is the percent of genomic DNA in mixed sample [25].

## Competing interests

The authors declare that they have no competing interests.

## Authors' contributions

JW performed bisulphite primer design, cloning and sequencing the target regions from genomic and bisulphite treated DNA in different species, cytosine methylation profile analysis, contributed extensively to the writing of the manuscript, and secured funds (20100480915). JM conceived the ideas, guided the data analysis and revised the manuscript. GK also conceived the ideas and advised JW for the experiments, critically read and improved the manuscript both in terms of academic content and expression of English. CH, SK and YL helped conceive the study and provided advice on development of control assays. CW collected plant samples for the analysis. KL critically read and revised the manuscript. All authors read and approved the final manuscript.

## Supplementary Material

Additional file 1 **Gene information for primer design**. Click here for file
