# Supplementary material for: Universal endogenous gene controls for bisulphite conversion in analysis of plant DNA methylation
Source: Plant Methods. 2011 Dec 2;7:39. doi: 10.1186/1746-4811-7-39 (PMC3260233; doi:10.1186/1746-4811-7-39)
Supplement: Additional file 1 — Gene information for primer design. [file 1746-4811-7-39-S1.PDF]

Species	Locus	GenBank Accession No.
Brassica napus	IND.a_A3	HB416515
	IND.a_C3	HB416517
	ATP1	AP006444
	NAD1	AP006444
Brassica juncea	ATP1	FJ626737
Brassica rapa	IND.a_A3	GU386357
	ATP1	AF076166
	NAD1	DX906492
	ATS1_A1	http://brassicadb.org/brad/
Brassica oleracea	IND.a_C3	GU386356
	ATP1	BH737254
	NAD1	BH678312
Arabidopsis thaliana	ATP1	Y08501
	NAD1	Y08501
Oryza sativa	ATP1	AP011077
